# *gnas* Knockdown Induces Obesity and AHO Features in Early Zebrafish Larvae

**DOI:** 10.3390/ijms252312674

**Published:** 2024-11-26

**Authors:** Alaa Abbas, Ayat S Hammad, Zain Z. Zakaria, Maha Al-Asmakh, Khalid Hussain, Mashael Al-Shafai

**Affiliations:** 1 Department of Biomedical Sciences, College of Health Sciences, QU Health, Qatar University, Doha P.O. Box 2713, Qatar; aa1604042@qu.edu.qa (A.A.); ayat.hammad@qu.edu.qa (A.S.H.); zain.zakaria@qu.edu.qa (Z.Z.Z.); maha.alasmakh@qu.edu.qa (M.A.-A.); 2 Biomedical Research Center, Qatar University, Doha P.O. Box 2713, Qatar; 3 Division of Endocrinology, Department of Pediatric Medicine, Sidra Medicine, Doha P.O. Box 26999, Qatar; khussain@sidra.org

**Keywords:** *GNAS* (Guanine Nucleotide-Binding Protein, Alpha Stimulating), knockdown, monogenic obesity, Morpholino, zebrafish model

## Abstract

*GNAS* (Guanine Nucleotide-Binding Protein, Alpha Stimulating) is a complex gene that encodes the alpha subunit of the stimulatory G protein (G_s_α), critical for signaling through various G protein-coupled receptors. Inactivating genetic and epigenetic changes in *GNAS*, resulting in G_s_α deficiency, cause different variants of pseudohypoparathyroidism, which may manifest features of Albright hereditary osteodystrophy (AHO, a syndrome characterized by early-onset obesity and other developmental defects). Recent findings have linked G_s_α deficiency with isolated, severe, early-onset obesity, suggesting it as a potential, underrecognized cause of monogenic, non-syndromic obesity. This study was prompted by identifying several *GNAS* variants of uncertain significance (VUSs) in pediatric patients presenting with unexplained, severe, early-onset obesity at Sidra Medicine in Qatar. To functionally characterize these variants, we developed the first zebrafish model of G_s_α deficiency, offering numerous advantages over other model systems. This was achieved by knockdown of the ortholog through microinjection of translation-blocking Morpholino antisense oligonucleotides into the yolks of 1-8-cell-stage zebrafish embryos. The morphant larvae displayed an obese phenotype, marked by significantly enlarged yolk sacs, increased neutral lipid accumulation, and reduced metabolic rates, among other developmental abnormalities resembling those in AHO. This zebrafish model lays the foundation for efficient functional characterization of *GNAS* VUSs and paves the way for enhancing our understanding of G_s_α deficiency-associated early-onset obesity.

## 1. Introduction

Early-onset obesity, defined as obesity occurring in children aged 5 years and below, represents a significant global health problem [1]. As reported by the World Health Organization, approximately 39 million children under the age of 5 years were overweight or obese in 2020 [2]. Childhood obesity is particularly prevalent in the Middle East and North Africa (MENA) region [3]. A notable example is Qatar, where about 50% of school-aged children are classified as overweight or obese [4]. The consequences of early-onset obesity extend far beyond increased body weight; it is linked to several comorbidities and complications, including type 2 diabetes mellitus, cardiovascular diseases, polycystic ovarian syndrome, sleep apnea, psychological problems, and musculoskeletal disorders [5].

This condition is classified into distinct forms based on its etiology and clinical spectrum, namely, polygenic, monogenic, and syndromic [6]. The polygenic form is the most prevalent, resulting from complex interactions between multiple genetic and environmental factors [7]. Conversely, the monogenic form is rare, primarily caused by autosomal recessive variants in single genes, often associated with the leptin-melanocortin pathway, which regulates energy homeostasis and appetite [8]. It presents as severe, early-onset obesity, indicated by a body mass index (BMI) that is at least 120% of the 95th age- and sex-specific percentile [1], and is frequently associated with endocrine disorders and hyperphagia [9]. Given the high prevalence of consanguinity [10], monogenic obesity poses a significant health concern in Arab populations [11], underscoring the need for more research efforts to explore the genetic basis of obesity in the region [12]. Lastly, the syndromic form arises from various genetic factors, involves multiple organ systems, and is associated with additional clinical features, such as developmental and hormonal abnormalities [6].

*GNAS* (Guanine Nucleotide-Binding Protein, Alpha Stimulating) is an imprinted, complex gene located on chromosome 20 that encodes the alpha subunit of the heterotrimeric stimulatory G protein (G_s_α) [13]. This protein subunit mediates signaling through various seven-transmembrane G protein-coupled receptors (GPCRs), including the melanocortin 4 receptor (MC4R), which plays a crucial role in regulating energy homeostasis, food intake, and body weight [14]. Genetic or epigenetic alterations in *GNAS* that lead to G_s_α deficiency are known to cause pseudohypoparathyroidism (PHP) in its different subtypes [15]. PHP refers to a group of rare disorders characterized by resistance to parathyroid hormone (PTH), among other clinical symptoms [16]. Some PHP subtypes exhibit features of Albright hereditary osteodystrophy (AHO), a rare syndrome that includes early-onset obesity, short stature, skeletal anomalies, developmental delay, and mental development issues [17]. More recently, G_s_α deficiency has been linked to isolated, severe, early-onset obesity, with accumulating evidence suggesting that it may be an underappreciated cause of monogenic, non-syndromic obesity [18,19].

To delineate the molecular mechanisms underlying G_s_α deficiency-associated early-onset obesity, various functional studies have been conducted in mice, where the *Gnas* gene was manipulated to model G_s_α deficiency [20,21,22,23]. Although the mouse model has significantly advanced our understanding of the disease pathogenesis, much remains to be explained [23]. Importantly, the mouse model has notable limitations, including its frequent inability to accurately model human diseases due to the differences between humans and mice in the processes that link genetic changes to disease development [24]. Additionally, mouse models are expensive, complicated, and time-consuming to generate and study [25]. Therefore, there is a pressing need for an improved and more robust model organism to facilitate further studies of the pathophysiology of early-onset obesity associated with *GNAS* changes. This would accelerate the discovery of novel therapies and enable the functional characterization of *GNAS* variants of uncertain significance (VUSs), ultimately improving patient outcomes.

The zebrafish (*Danio rerio*) has become an increasingly valuable model for studying various human metabolic disorders, including obesity and diabetes [26]. This is primarily due to the remarkable similarity and functional conservation of the main organs and metabolic pathways between zebrafish and humans [27]. Zebrafish have proven to be an excellent model for human developmental and genetic diseases owing to their multiple advantageous features [28]. Notably, zebrafish are approximately 70% genetically similar to humans, sharing over 80% of human disease-associated genes [29]. This model organism is easy to manipulate genetically, produces large numbers of offspring, is cost-effective and easy to maintain, and has a fully sequenced genome [30]. Moreover, its rapid and external development and the transparency of its embryonic and larval stages make it easy to manipulate and study [31]. Furthermore, this vertebrate is anatomically and physiologically similar to humans, making it a suitable animal model for human diseases [32].

Despite the development of multiple zebrafish obesity models, including diet-induced models and various transgenic and mutant lines [27], early-onset obesity induced by G_s_α deficiency has yet to be modeled in zebrafish. Thus, owing to its numerous advantages, we sought to develop the first zebrafish model of this specific condition. Utilizing Morpholino-mediated knockdown of the *gnas* gene, we examined the effects of G_s_α deficiency in the zebrafish during the embryonic and early larval stages (up to 5 days post-fertilization). Our study focused on various metabolic, morphometric, and developmental parameters to establish the loss-of-function phenotype in zebrafish and provide novel insights into the disease pathophysiology. This zebrafish model lays the groundwork for further functional studies, potentially enabling the efficient reclassification of novel and previously reported *GNAS* VUSs.

## 2. Results

### 2.1. Gα_sS_ Temporal Expression Analysis in the Zebrafish

**Figure 1:**
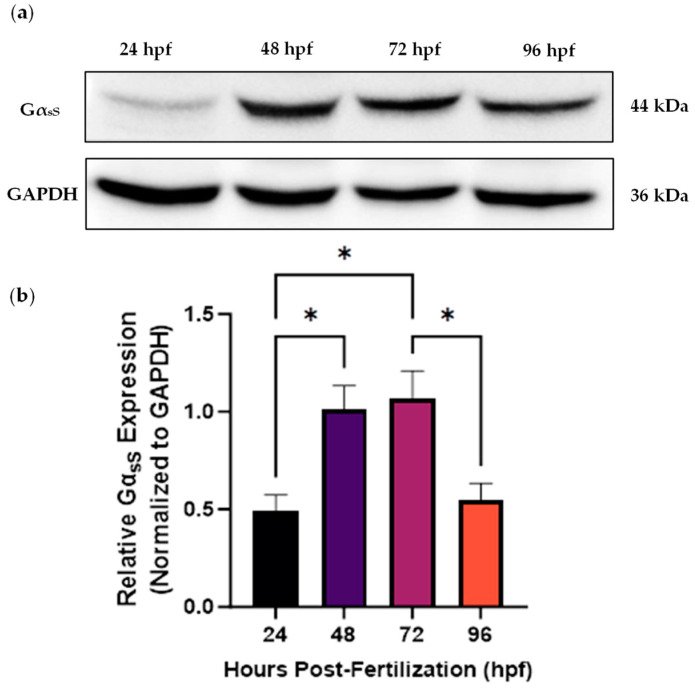
Western blot analysis of the temporal expression pattern of the short Gα_s_ isoform (Gα_sS_) in whole wild-type zebrafish from 24 to 96 h post-fertilization (hpf). (**a**) Representative blot of the Gα_sS_ expression level using GAPDH as a loading control. Whole zebrafish tissue lysates were used to extract total protein (~40 µg loaded per well). (**b**) The band intensities were quantified using ImageJ software, followed by normalization of the Gα_sS_ band intensity to that of GAPDH, yielding the relative Gα_sS_ expression level in arbitrary units. One-way ANOVA followed by Tukey’s multiple comparisons test were performed (* *p* < 0.05). Each bar represents the mean ± SEM (*n* = 3).

Prior to the knockdown experiments, the temporal expression pattern of the protein of interest, specifically the short Gα_s_ isoform (Gα_sS_) encoded by *gnas*, was analyzed by Western blotting in whole zebrafish embryos and larvae at 24–96 hpf. This analysis was performed to determine the appropriate timepoint at which to assess the knockdown efficiency of the translation-blocking Morpholinos. There is at least one other Gα_s_ splice variant expressed in zebrafish, known as the long Gα_s_ isoform (Gα_sL_; 49 kDa) [33]. However, its expression level is much lower compared to Gα_sS_. Consequently, Gα_sS_ was selected as the isoform of interest. The results revealed that Gα_sS_ expression increased significantly from 24 hpf until 72 hpf (1.07 a.u. at 72 hpf vs. 0.49 a.u. at 24 hpf; *p* < 0.05), followed by a significant decrease to 0.55 a.u. at 96 hpf (*p* < 0.05) (Figure 1). Based on these findings and considering the diminishing effectiveness of Morpholinos over time [34], 48 hpf was chosen as a suitable timepoint to analyze knockdown efficacy. Importantly, we tested different commercially available antibodies during this stage and successfully validated the anti-GNAS antibody (Abcam, Cambridge, UK; catalog # ab97629), targeting the human G_s_α protein, for use with zebrafish samples. This represents the first validation of this antibody for zebrafish, enhancing its utility for future research.

### 2.2. Determination of Optimal Morpholino Dose for gnas Gene Knockdown in Zebrafish

To block the translation of the two G_s_α splice variants in zebrafish, two different Morpholino oligomers (MOs), each targeting one of the splice variants, were simultaneously microinjected into the yolks of 1-8-cell-stage embryos (Table 1). Three doses (1, 3, and 5 ng) of both MOs mixed were tested for toxicity and efficacy to identify the optimal dose. These doses were selected based on reports that MO doses of 5 ng or less result in specific loss-of-function phenotypes with minimal off-target effects and toxicity [35,36,37].

#### 2.2.1. Knockdown Efficacy at 48 Hours Post-Fertilization

**Figure 2:**
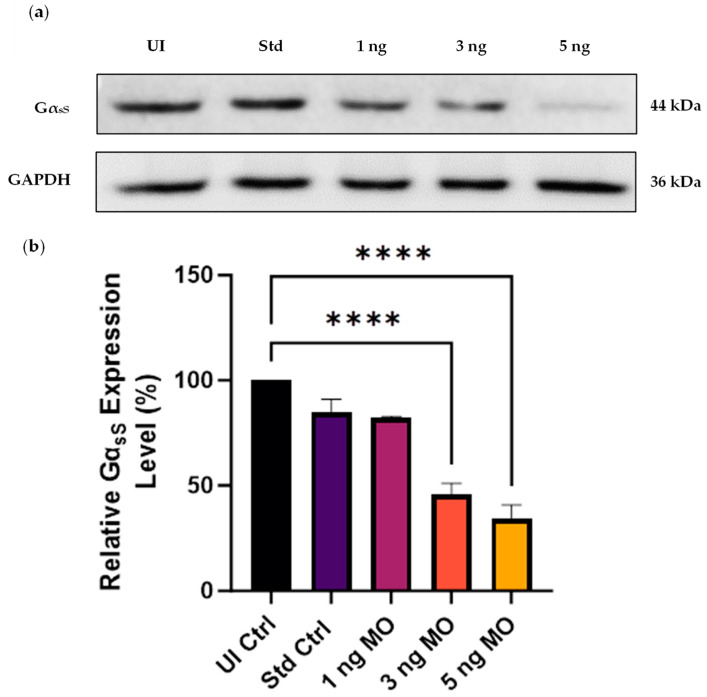
Western blot analysis of the knockdown efficiency of the three *gnas* Morpholino (MO) doses at 48 hpf. (**a**) Representative blot of the Gα_sS_ expression level in non-injected control (UI Ctrl) and standard control (Std Ctrl) embryos compared to those injected with 1, 3, and 5 ng of *gnas* MOs. The Std Ctrl embryos were injected with 5 ng of Std Ctrl MO. Whole zebrafish tissue lysates were used to extract total protein (~40 µg loaded per well) and GAPDH served as a loading control. (**b**) The band intensities were quantified using ImageJ software, followed by normalization of the Gα_sS_ band intensity to that of GAPDH. The Gα_sS_ expression level was expressed as a percentage of that in the UI Ctrl embryos. One-way ANOVA followed by Tukey’s multiple comparisons test were performed (**** *p* < 0.0001). Each bar represents the mean ± SEM (*n* = 4).

The level of *gnas* knockdown was quantified at the protein level by Western blotting at 48 hpf to determine the knockdown efficiency of the three MO doses (1, 3, and 5 ng). Compared to non-injected controls, the decrease in expression level was statistically significant at MO doses of 3 ng and 5 ng (*p* < 0.0001). The 5 ng *gnas* MO dose resulted in the highest level of knockdown, causing approximately 66% reduction in Gα_sS_ expression level (Figure 2).

#### 2.2.2. Toxicity Assessment of the Morpholino Doses

**Figure 3:**
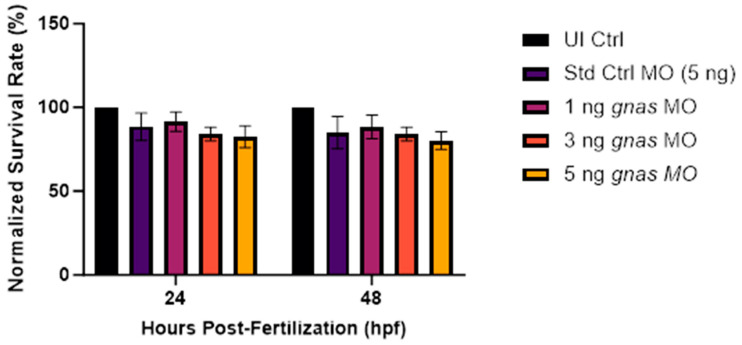
The survival rates at 24 and 48 hpf of the zebrafish embryos (*n* ≈ 20 per group) injected with 5 ng of Std Ctrl MO or different doses (1, 3, or 5 ng) of *gnas* MOs. The survival rates were normalized to those of the non-injected controls (UI Ctrl). Two-way ANOVA and Tukey’s post hoc test were performed, indicating no significant differences between the survival rates at either timepoints. Each bar represents the mean ± SEM (*n* = 4).

To assess the toxicity of the different *gnas* MO doses, the survival, tail-flicking, and hatching rates of the morphant zebrafish embryos were measured and compared to those of non-injected controls and negative controls injected with 5 ng of Std Ctrl MO. Firstly, the survival rate was recorded every 24 hpf until the experimental endpoint, which was 48 hpf in this first phase of this study that aimed to determine the optimal MO dose for *gnas* knockdown in zebrafish embryos. The survival rates of all injected groups were expressed as a percentage of that of the non-injected control group. There was no significant difference between the mean survival rates of the different experimental and control groups (*n* = 4; Figure 3). However, a dose-dependent decrease in survival rate was observed in the groups injected with the different doses of *gnas* MOs, with the lowest survival rates (83% and 80% at 24 and 48 hpf, respectively) seen in the group injected with the highest dose of 5 ng. Nevertheless, this decrease in survival was statistically insignificant (Figure 3). 

**Figure 4:**
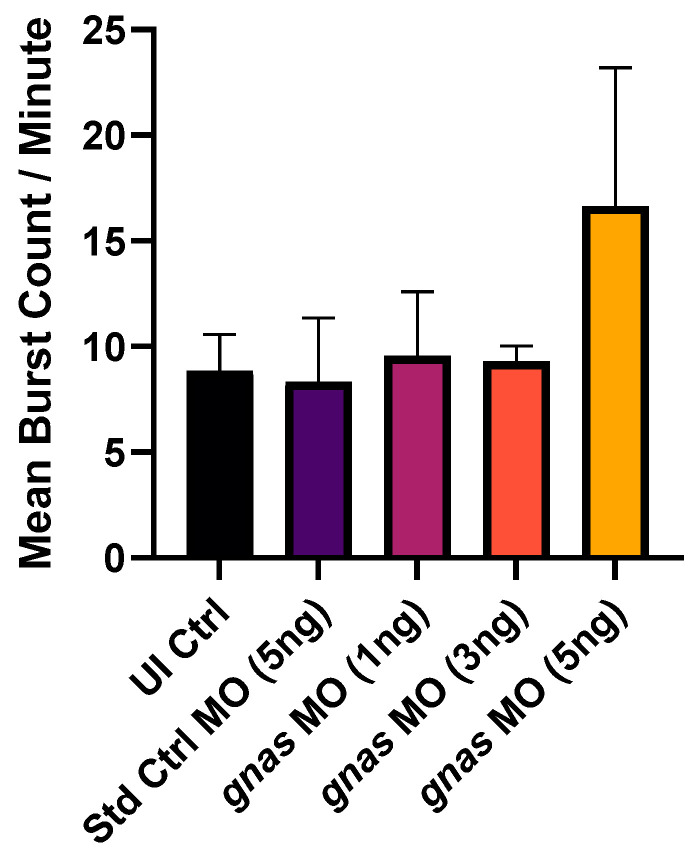
The rate of tail flicks at 24 hpf of the zebrafish embryos (*n* ≈ 10 per group) injected with 5 ng of Std Ctrl MO or different doses (1, 3, or 5 ng) of *gnas* MOs, compared to non-injected controls (UI Ctrl). The frequency of this spontaneous movement was measured by the DanioScope software as the mean burst count per minute. One-way ANOVA and Tukey’s multiple comparisons test were performed, indicating no significant differences between the mean tail-flicking rates. Each bar represents the mean ± SEM (*n* = 4).

Secondly, the rate of spontaneous tail flicks of zebrafish embryos at 24 hpf was measured as an indicator of neurotoxicity using the DanioScope software [38]. Tail flicking is the first movement elicited by the developing nervous system of zebrafish embryos [39]. Our analysis revealed no significant differences in the tail-flicking rates, represented by the mean burst count per minute, among the different injected and non-injected groups (Figure 4). However, the frequency of tail flicks was higher, yet more variable, in the group injected with 5 ng of *gnas* MOs compared to the other groups (Figure 4).

**Figure 5:**
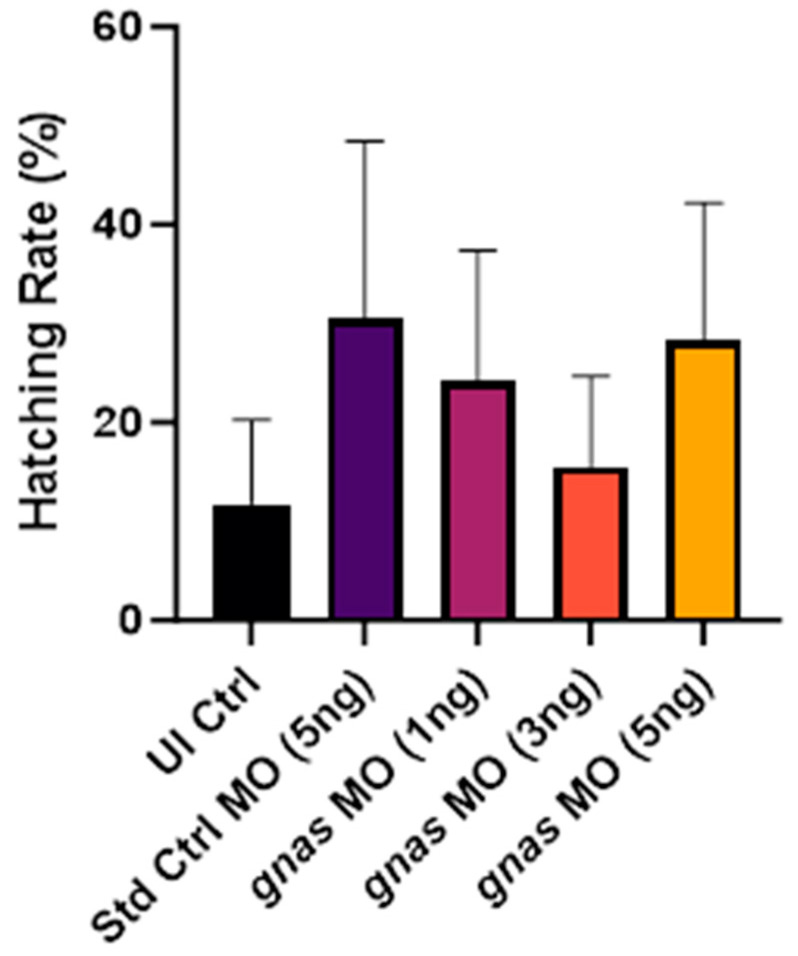
The hatching rates at 48 hpf of the zebrafish embryos (*n* ≈ 20 per group) injected with 5 ng of Std Ctrl MO or different doses (1, 3, or 5 ng) of *gnas* MOs, compared to non-injected controls (UI Ctrl). One-way ANOVA and Tukey’s multiple comparisons test were performed, indicating no significant differences between the mean hatching rates. Each bar represents the mean ± SEM (*n* = 4).

Finally, the embryo-hatching rate was recorded at 48 hpf, as it may serve as an indicator of developmental toxicity, where a delay in hatching could reflect developmental delay [39]. The normal zebrafish hatching period is between 48 and 72 hpf [40]. The hatching rates across all groups exhibited substantial variability, as indicated by the large error bars in Figure 5. This variability will be further discussed in Section 3. Overall, no statistically significant differences existed between the mean hatching rates of all experimental and control groups.

### 2.3. Phenotypic Analyses of gnas Morphants

To assess the dose-dependent specific effects of MO-mediated *gnas* knockdown on different phenotypes related to G_s_α deficiency and obesity, zebrafish embryos injected with 3 ng and 5 ng of *gnas* MOs were evaluated for their yolk sac areas, body lengths, body weights, neutral lipid contents, and hatching rates (at 72 hpf), which were compared to those of non-injected and Std Ctrl MO-injected controls.

#### 2.3.1. *gnas* Knockdown Increased Neutral Lipid Content and Yolk Sac Size in Zebrafish Larvae at 120 hpf

The amounts of neutral lipids in the zebrafish larvae were quantified at 120 hpf by Oil Red O (ORO) staining of the whole larvae, followed by extraction of the stain from the zebrafish bodies and measurement of the absorbance at 495 nm. ORO staining was performed at this specific timepoint to capture the largest possible differences in neutral lipid content between experimental groups, as resorption of the lipid-rich yolk becomes evident around 120 hpf during normal development [41,42]. In addition, previous studies have performed ORO staining at this stage and shown that lipids accumulate in many tissues and organs at 120 hpf [43,44].

**Figure 6:**
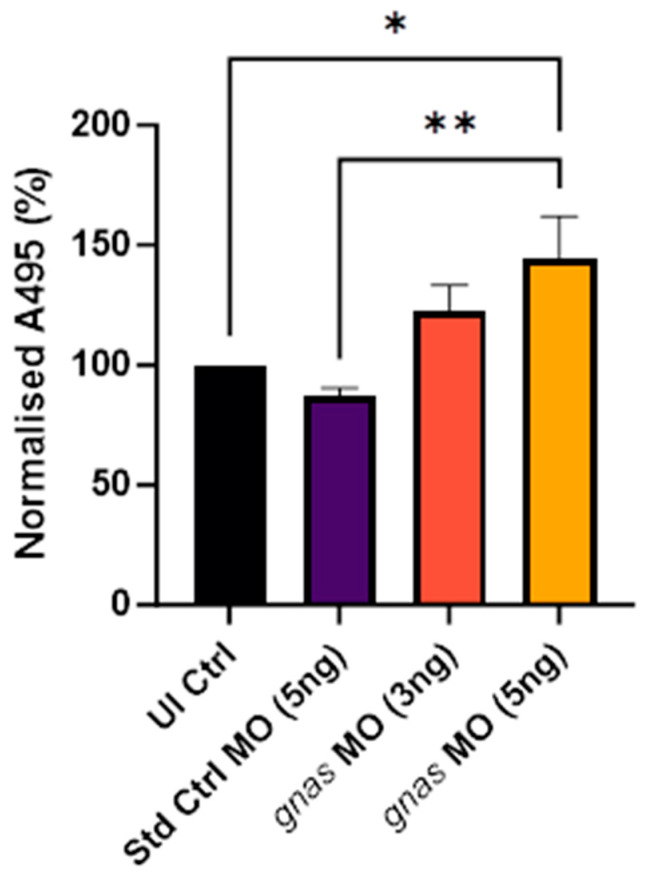
Quantification of the Oil Red O staining at 120 hpf of whole zebrafish larvae (*n* ≈ 20 per group) injected with 5 ng of Std Ctrl MO or two doses (3 or 5 ng) of *gnas* MOs. Staining was quantified by the absorbance of the extracted stain at 495 nm and normalized to that of non-injected controls (UI Ctrl). One-way ANOVA and Tukey’s multiple comparisons test were performed (* *p* < 0.05 and ** *p* < 0.01). Each bar represents the mean ± SEM (*n* = 5).

A dose-dependent increase in the absorbance (A495) was observed in the *gnas* morphants compared to the controls, indicating an increase in neutral lipid content (Figure 6). This change was statistically significant at the higher (5 ng) *gnas* MO dose relative to the UI Ctrl and Std Ctrl groups (*p* < 0.05 and *p* < 0.01, respectively). This is likely a specific effect of *gnas* knockdown, as Std Ctrl morphants did not exhibit an increase in staining, but rather a slight, insignificant decrease compared to non-injected controls.

The yolk of the zebrafish embryo, rich in lipids and proteins, is a site of active lipid metabolism, where lipid breakdown and synthesis occur before mobilization to the embryo [45]. An increase in yolk size, known as yolk retention, indicates impaired lipid metabolism and uptake [46]. Yolk sac size was measured at 72 and 120 hpf, as described in a previous study [47], to assess differences in yolk consumption across groups at both timepoints.

**Figure 7:**
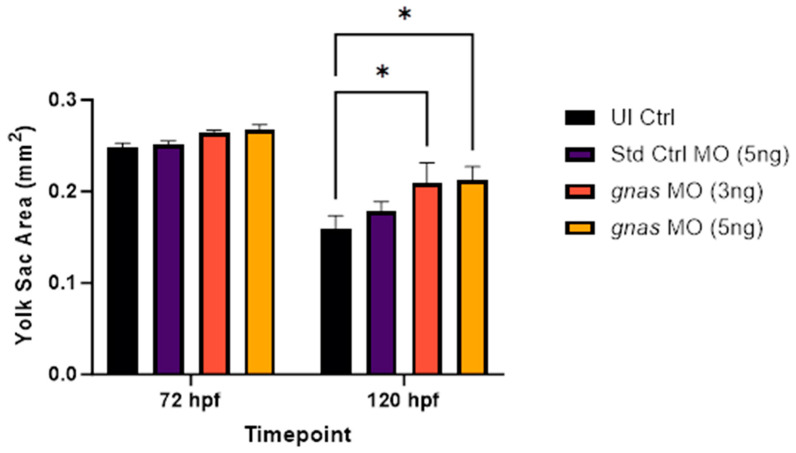
Yolk sac areas of zebrafish larvae (*n* = 10 per group), at 72 and 120 hpf, injected with 5 ng of Std Ctrl MO or two doses (3 or 5 ng) of *gnas* MOs, compared to those of non-injected controls (UI Ctrl). Two-way ANOVA and Tukey’s multiple comparisons test were performed, indicating significant increases in yolk size of *gnas* morphants at 120 hpf compared to non-injected controls (* *p* < 0.05). Each bar represents the mean ± SEM (*n* = 3).

Knockdown of the *gnas* gene resulted in a significant increase in yolk sac area in zebrafish larvae at 120 hpf, compared to non-injected controls (*p* < 0.05), as depicted in Figure 7. At 72 hpf, slight but insignificant increases in yolk sac areas were noted in *gnas* morphants compared to non-injected controls. No significant difference was observed between the mean yolk size of Std Ctrl morphants and that of non-injected controls, suggesting that this effect was specific to *gnas* knockdown.

#### 2.3.2. *gnas* Knockdown Led to a Significant, Specific Reduction in Metabolic Rate

**Figure 8:**
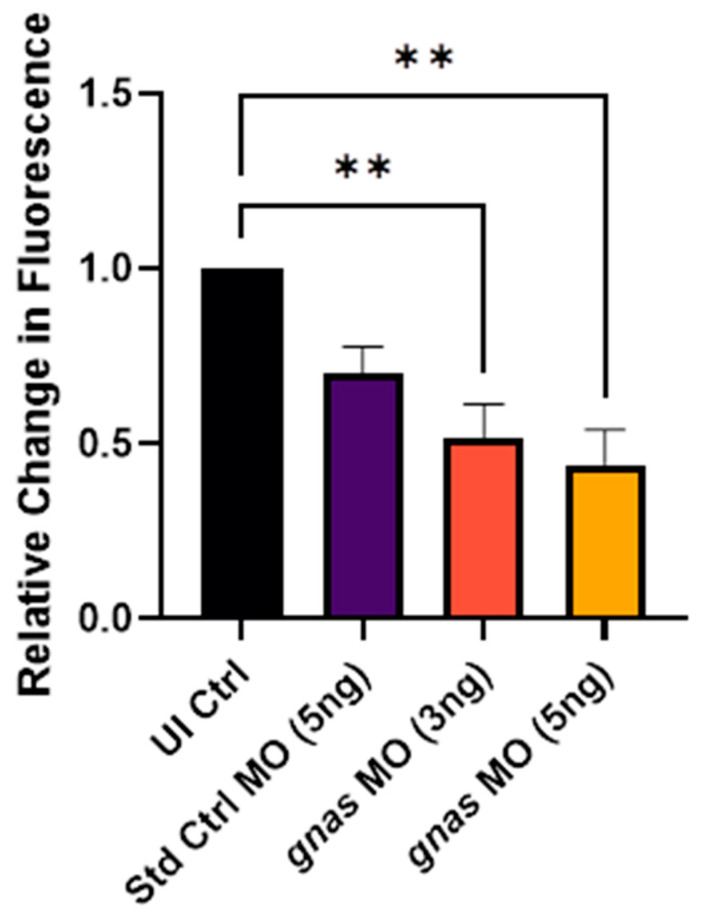
Relative change in fluorescence after incubation in alamarBlue^TM^ assay buffer of zebrafish larvae injected with 5 ng of Std Ctrl MO or two doses (3 or 5 ng) of *gnas* MOs, normalized to that of non-injected controls (UI Ctrl). The larvae (*n* ≈ 12 per group) were incubated from 72 to 96 hpf. One-way ANOVA and Tukey’s multiple comparisons test were performed (** *p* < 0.01). Each bar represents the mean ± SEM (*n* = 4).

The alamarBlue^TM^ assay, which measures in vivo NADH_2_ production via the Krebs cycle as a direct indicator of metabolic rate, was performed at 72–96 hpf based on previous studies [48,49]. In this assay, the non-fluorescent compound resazurin permeates the zebrafish tissues, where it is reduced by NADH_2_ in the metabolically active cells to produce the fluorescent compound resorufin [48]. Compared to non-injected controls, *gnas* morphants exhibited significantly lower metabolic rates, as indicated by the relative change in fluorescence from 72 to 96 hpf (*p* < 0.01). This decrease was greatest for the morphants injected with 5 ng of *gnas* MOs, with a relative change in fluorescence of 0.44 (*p* < 0.01), and smallest for the morphants injected with 5 ng of Std Ctrl MO, which displayed a relative change in fluorescence of 0.70 (ns; Figure 8). Thus, this effect was MO-dose-dependent, statistically significant, and specific to *gnas* knockdown.

#### 2.3.3. *gnas* Morphants Exhibited Skeletal Abnormalities and Reduced Body Lengths

**Figure 9:**
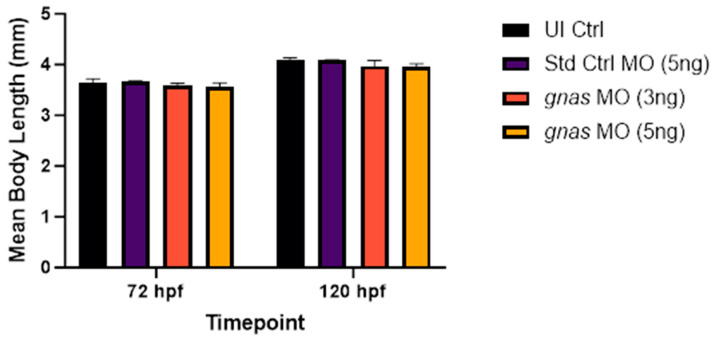
The mean body lengths of zebrafish larvae (*n* = 10 per group), at 72 and 120 hpf, injected with 5 ng of Std Ctrl MO or two doses (3 or 5 ng) of *gnas* MOs, compared to those of non-injected controls (UI Ctrl). Two-way ANOVA and Tukey’s multiple comparisons test were performed, indicating no significant differences. Each bar represents the mean ± SEM (*n* = 3).

**Figure 10:**
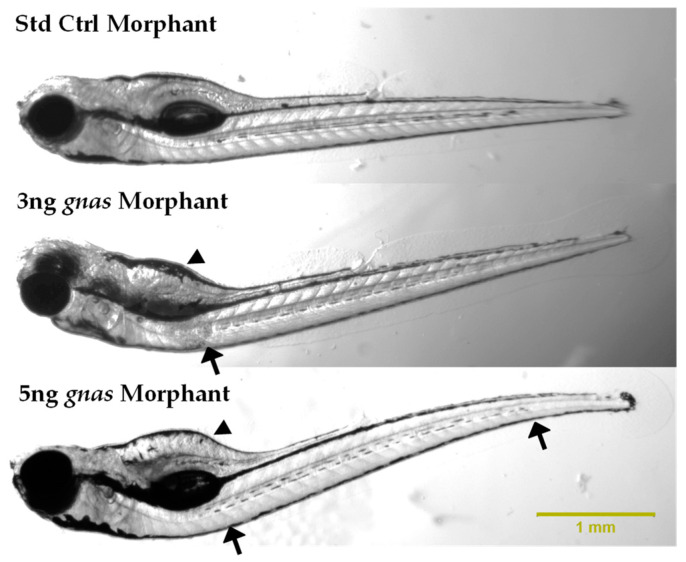
Representative images of the lateral views of standard control and *gnas* (3 and 5 ng) morphants at 120 hpf. Spine curvature and yolk sac enlargement of the *gnas* morphants are indicated by arrows and arrowheads, respectively. The images were captured at 20× magnification following straightening of the larvae in methylcellulose as much as possible.

The body lengths of the zebrafish morphants were measured using ImageJ software to assess the effect of *gnas* knockdown on growth and skeletal development, considering the association of G_s_α deficiency with skeletal defects and short stature in humans [18]. This was performed at 72 and 120 hpf, as described by Shah et al. (2019) [47]. The results showed that *gnas* morphants exhibited slightly reduced body lengths at 72 and 120 hpf compared to controls. However, this trend was not statistically significant (Figure 9). Additionally, morphological assessments revealed a greater incidence of skeletal abnormalities, such as scoliosis and tail curvature, in *gnas* morphants compared to controls (Figure 10).

#### 2.3.4. *gnas* Morphants Showed a Slight but Non-Specific Increase in Mean Larval Wet Mass

**Figure 11:**
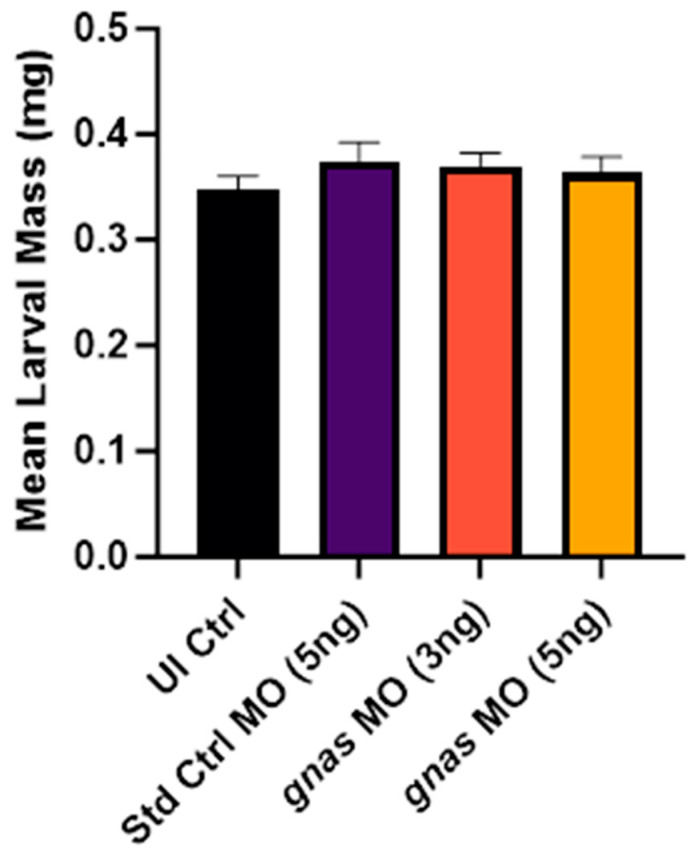
The mean larval masses of zebrafish larvae (*n* ≈ 20 per group), at 120 hpf, injected with 5 ng of Std Ctrl MO or two doses (3 or 5 ng) of *gnas* MOs, compared to those of non-injected controls (UI Ctrl). One-way ANOVA and Tukey’s multiple comparisons test were performed, indicating no significant differences. Each bar represents the mean ± SEM (*n* = 3).

In the investigation of larval mass at 120 hpf, wet masses of zebrafish larvae were quantitatively assessed using a high-precision analytical balance. This timepoint was chosen for this assessment to facilitate larval mass measurement, as it was the final timepoint before euthanasia and the larvae were at their largest. The results indicated that both *gnas* and Std Ctrl morphants had slightly increased average larval masses compared to non-injected controls, although this was not statistically significant (Figure 11). This may be explained by the challenges associated with the measurement technique, which are further discussed in Section 3.1.

#### 2.3.5. *gnas* Knockdown Resulted in Delayed Hatching at 72 hpf

**Figure 12:**
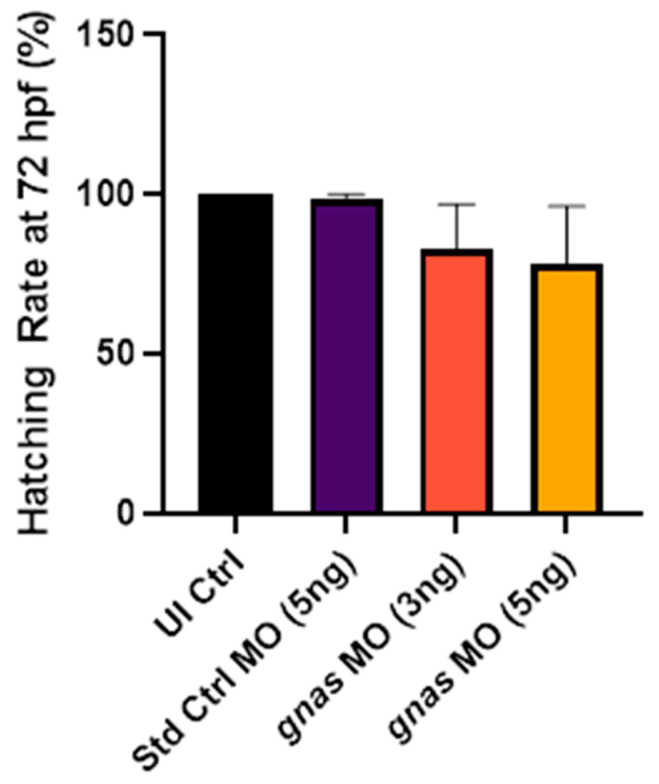
Hatching rates at 72 hpf of zebrafish larvae (*n* ≈ 25 per group) injected with 5 ng of Std Ctrl MO or two doses (3 or 5 ng) of *gnas* MOs, compared to those of non-injected controls (UI Ctrl). One-way ANOVA and Tukey’s multiple comparisons test were performed, indicating no significant differences. Each bar represents the mean ± SEM (*n* = 3).

The hatching rate of zebrafish larvae at 72 hpf may indicate their development rate. *gnas* morphants exhibited delayed hatching at this timepoint compared to non-injected controls and Std Ctrl morphants, suggesting developmental delay due to *gnas* knockdown. However, this effect was not statistically significant given the relatively large variability in the hatching rates of the *gnas* morphants, as indicated by the error bars in Figure 12.

#### 2.3.6. *gnas* Morphants Manifested Largely Increased Triglyceride Levels with No Appreciable Changes in cAMP and Leptin Levels

**Figure 13:**
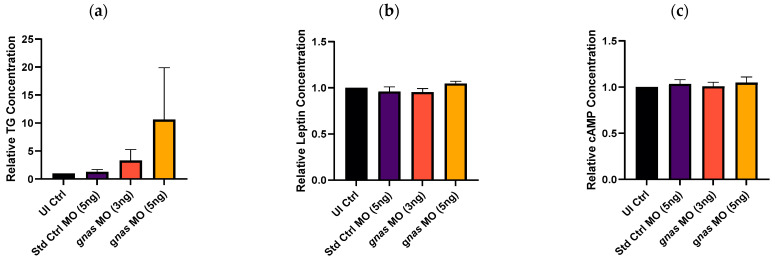
Concentrations of different molecules in whole zebrafish larvae at 72 hpf, determined by ELISA. (**a**) Triglyceride, (**b**) leptin, and (**c**) cAMP levels in larvae (*n* ≈ 35 per group) injected with 5 ng of Std Ctrl MO or two doses (3 or 5 ng) of *gnas* MOs, relative to those of non-injected controls (UI Ctrl). One-way ANOVA and Tukey’s multiple comparisons test were performed, indicating no significant differences. Each bar represents the mean ± SEM (*n* = 3).

ELISA was employed to assess the triglyceride (TG), cAMP, and leptin levels in *gnas* morphant larvae compared to controls. This was performed at 72 hpf as it was an appropriate timepoint as described in the literature [50]. The results indicated a dose-dependent increase in TG levels specific to the *gnas* morphants, while the cAMP and leptin levels remained largely unaffected. Despite these observations, the increase in TG level did not show statistical significance due to the large variability in the data, particularly for the morphants injected with 5 ng of MOs, as indicated by the large error bar (Figure 13). This variability may be due to various reasons, including variability in kit age and gene knockdown efficiency across different embryos within the same experimental group.

## 3. Discussion

In the current study, we characterized the impacts of *gnas* gene knockdown on various parameters in early zebrafish larvae to establish the loss-of-function phenotype. This investigation provides significant insights into the roles of G_s_α deficiency in inducing obesity, metabolic dysfunction, and developmental abnormalities. The knockdown of the zebrafish *gnas* gene was effectively achieved through the microinjection of translation-blocking MOs into the yolks of 1-8-cell-stage embryos. The embryos and larvae were closely monitored for 5 days post-fertilization. During this time, comprehensive assessments of several metabolic, morphometric, and developmental parameters were made, establishing the first zebrafish knockdown model of G_s_α deficiency.

In our research, MO doses of 5 ng or lower were employed, consistent with research reports that these concentrations generally induce specific loss-of-function phenotypes with minimal toxicity and off-target effects [35,36,37]. Among the dosages tested in our experiments, 5 ng was identified as the optimal concentration due to its superior efficacy and low toxicity (Figure 2). The specificity of the *gnas* MO-induced knockdown was confirmed by the modest 15% reduction in Gα_sS_ expression observed with the 5 ng dose of the Std Ctrl MO.

The specificity of the *gnas* knockdown facilitated a focused examination of different phenotypes in the early zebrafish embryos. With respect to the hatching rate at 48 hpf (Figure 5), the large variability could be attributed to differences in the quality of embryos from distinct clutches and breeding tanks [51]. It is also noted that the hatching times can vary significantly even within a single clutch and that earlier hatching does not invariably suggest faster development [52].

With regard to the analysis of tail-flicking rates at 24 hpf, the group injected with the 5 ng dose of *gnas* MOs exhibited the highest but most variable rates of tail flicking (Figure 4). Although this increase in the frequency of spontaneous tail flicks was statistically insignificant, it may reflect hyperactivity and epileptic-like movements, as described in a study by Basnet et al. (2017) [53]. This may be a direct consequence of *gnas* knockdown, as in humans with pseudohypoparathyroidism type 1A (PHP1A), where maternal G_s_α expression or function is impaired, as hypocalcemia was reported in several studies to be associated with neuromuscular disorders and seizures [15,18,54].

In this study, zebrafish *gnas* morphants exhibited an obese phenotype characterized by a significant increase in neutral lipid content, enlarged yolk sacs, and diminished metabolic rates. These observations, including neutral lipid accumulation and yolk retention observed at 120 hpf, together with the elevated TG level determined by ELISA at 72 hpf, suggest impaired lipid metabolism due to *gnas* knockdown. Neutral lipids present in the yolk, such as triglycerides and cholesterol, serve as a critical energy source during embryonic and early larval stages of zebrafish development [55,56]. The yolk undergoes active lipid processing and remodeling before these lipids are absorbed by the developing embryo [45]. The yolk syncytial layer, which is found between the yolk and blastoderm, is implicated in the hydrolysis of complex lipids and synthesis of lipoproteins that transport lipids to the embryo [57]. Thus, knockdown of *apoc2*, the gene encoding apolipoprotein C-II in zebrafish, was demonstrated to result in yolk retention due to impaired lipid metabolism [58]. During normal development, yolk resorption becomes apparent at around 120 hpf when exogenous feeding begins, with complete depletion occurring at approximately 170 hpf [41,42].

The observed increase in lipid content and yolk size in the *gnas* morphants may be a result of Gα_s_ deficiency, which, in humans, is associated with metabolic dysfunction [59]. Given the similarity between lipid metabolic pathways in humans and zebrafish, similar mechanisms may underly the lipid accumulation in *gnas* morphants and patients with PHP1A (which arises from maternal G_s_α deficiency) [60]. Notably, consistent with our findings, leptin A and leptin receptor zebrafish morphants exhibited enlarged yolk sacs and body/tail curvature, indicating the role of leptin-melanocortin signaling in lipid metabolism and early development [61].

Moreover, the *gnas* morphant zebrafish larvae exhibited significantly lower metabolic rates, slight reductions in body lengths, a higher incidence of skeletal deformities, particularly abnormal spine and tail curvature, and reduced hatching rates compared to the controls. These phenotypic manifestations closely resemble the features of AHO in humans, characterized by decreased energy expenditure, short stature, skeletal defects, and developmental delay. This syndrome is caused by heterozygous, loss-of-function variations in the *GNAS* gene [62]. These phenotypic parallels underscore the validity of the developed zebrafish model in mimicking human genetic disorders.

In humans, these characteristics arise from impaired GPCR signaling due to G_s_α deficiency, leading to hormone resistance [63,64]. For example, parathyroid hormone (PTH) resistance is linked to skeletal abnormalities and reduced growth in PHP1A patients, while growth hormone-releasing hormone (GHRH) resistance was associated with growth retardation and short stature [59,65]. Moreover, other studies showed that thyroid-stimulating hormone (TSH) resistance in PHP1A patients leading to hypothyroidism was linked to developmental delay, as normal thyroid function is critical for normal development [18,66]. Additionally, reduced energy expenditure in patients with G_s_α deficiency may be attributed to the impaired signaling of different GPCRs, such as β_2_- and β_3_-adrenergic receptors, thyroid-stimulating hormone receptor (TSHR), MC4R, and corticotropin-releasing hormone receptor (CRHR) [67]. Because GPCR signaling pathways are highly similar in zebrafish and humans, it is plausible that comparable mechanisms underly the observed phenotypes in the zebrafish *gnas* morphants [68].

Importantly, the reduced metabolic rate and increased lipid accumulation in our *gnas* morphant larvae, as determined by the alamarBlue^TM^ assay and ORO staining, respectively, were also observed in α-MSH mutant zebrafish larvae [49], demonstrating the importance of MC4R signaling in regulating energy expenditure and lipid metabolism. In addition, similar to *gnas* knockdown in zebrafish, *mc4r* knockout in medaka (*Oryzias latipes*) resulted in delayed hatching and reduced body length, suggesting a role of MC4R signaling in early development and growth [69].

### 3.1. Limitations

The present study faced a few methodological constraints. Firstly, the technique for measuring larval body mass at 120 hpf involved challenges that could have compromised measurement accuracy. The small size of the zebrafish larvae, coupled with the limited precision of the balance used and the difficulty in ensuring the complete removal of water droplets from the tube before weighing, might have contributed to the observed non-significant difference in mean wet larval mass across groups. This may also explain the apparent increase in body mass in the Std Ctrl morphants relative to the non-injected controls. Nevertheless, this method, which was developed after a thorough search of the literature, remains the most feasible one for weighing early zebrafish larvae based on our experience [70,71]. However, the results indicate that body mass may not be a reliable measure of adiposity at such an early larval stage. In contrast, neutral lipid staining, triglyceride level measurement by ELISA, and yolk sac size are more informative in this respect.

Additionally, the use of Morpholinos for gene knockdown had inherent limitations. Morpholinos do not achieve complete gene silencing, often produce off-target effects, and their efficacy diminishes over time as they are diluted through cellular processes [72]. Despite these issues, such limitations were not detrimental to the core objectives of our study. The goal was to establish a transient model of G_s_α deficiency (given the focus on early-onset obesity), where 50% knockdown was sufficient to simulate the 50% decrease in G_s_α activity in PHP1A [73]. To mitigate potential off-target effects, a standard control Morpholino was employed across all experiments, providing a baseline control to enhance the interpretability of the phenotypic changes observed [74].

### 3.2. Implications and Future Directions

Our study has successfully achieved its aims of developing and validating the first zebrafish model of G_s_α deficiency-associated early-onset obesity. In doing so, we have determined the optimal MO dose for *gnas* knockdown, in addition to testing for the first time an existing antibody (Abcam, Cambridge, UK, catalog # ab97629) designed to react with human G_s_α protein, proving that it reacts with the zebrafish Gα_sS_ protein. This will be of great use to researchers who wish to use this zebrafish model for further studies of G_s_α deficiency, which occurs in different subtypes of PHP, like PHP1A and pseudopseudohypoparathyroidism (PPHP). With its known advantages, this model will facilitate the study of the mechanisms underlying the various consequences of impaired G_s_-coupled receptor signaling, such as monogenic obesity, AHO, and hormone resistance, potentially accelerating the discovery of novel targeted therapies.

Looking ahead, future studies can utilize advanced techniques to delineate the effects of G_s_α deficiency on lipid metabolism in the zebrafish model. For example, the yolk sac may be dissociated from the zebrafish body to analyze their lipid compositions separately using mass spectrometry, as the yolk is the source of lipids, whereas the body is their destination [45]. Thus, treating the yolk and body as two independent systems would provide further insights into the changes in the lipid profile of the *gnas* morphants. Similarly, ORO staining of the yolk and body separately would shed light on the processes that are impaired as a result of G_s_α deficiency [75]. Moreover, fluorescent lipid analogs may be injected into the yolk to study any effect on lipid processing, and the expression of important genes involved in lipid metabolism during zebrafish development may be analyzed to elucidate the mechanisms responsible for lipid accumulation in the *gnas* morphants [45]. Further, transgenic fluorescent reporter zebrafish lines may be employed to better visualize specific organs, tissues, cells, or even molecules of interest, facilitating the study of disease pathophysiology [76].

In addition, different pharmacological treatments may be tested on this zebrafish model, including phosphodiesterase inhibitors (PDEIs) and receptor agonists (such as setmelanotide, a novel MC4R agonist recently approved for treating certain monogenic and syndromic obesity forms), expediting the development of novel therapies for use in humans. PDEIs, which inhibit the degradation of intracellular cAMP, thereby increasing its levels, may be promising for the treatment of G_s_α-deficiency-associated disorders, including early-onset obesity [77,78].

Another valuable use of the zebrafish *gnas* knockdown model is the functional characterization of novel *GNAS* variants and those of uncertain significance. This involves rescue experiments in the zebrafish morphants conducted by co-injection, with *gnas* MO, of wild-type or mutant complementary RNA (cRNA), corresponding to the wild-type human *GNAS* mRNA transcript and the transcript with the variant of interest, respectively. Briefly, after co-injecting different doses of wild-type human *GNAS* cRNA along with the optimal *gnas* MO dose, the optimal wild-type cRNA dose which results in the most significant rescue of the morphant phenotype with minimal toxicity is determined. This is followed by the ‘mutant rescue’ experiments, where the optimal dose (as determined for the wild-type cRNA) of the human *GNAS* mutant cRNA is injected into the zebrafish embryos along with the optimal MO dose, and phenotypic analyses are conducted as in the present study. The results of the wild-type and mutant rescue experiments are compared, enabling the functional characterization of the variant as described by Niederriter et al. (2013) [79].

## 4. Materials and Methods

### 4.1. Zebrafish Husbandry and Ethical Compliance

Wild-type (AB strain) zebrafish (*Danio rerio*) embryos were obtained from our zebrafish facility at the Biomedical Research Center at Qatar University. Adult zebrafish were housed in a recirculating stand-alone aquatic rack system (Aquaneering, San Diego, CA, USA; catalog # ZD560). The system was maintained under standard conditions: water temperature of 28 °C, a photoperiod regime of 14 h light and 10 h dark, and optimum water quality parameters [52]. The embryos were incubated at 28.5 °C in egg water (5 mM NaCl, 0.17 mM KCl, 0.16 mM MgSO_4_-7H_2_O, 0.4 mM CaCl_2_-2H_2_O, and 0.1% (*w*/*v*) methylene blue), which was replenished daily. All experiments were performed in compliance with national and international guidelines and approved by the Qatar University Institutional Biohazard Committee (reference number: QU-IBC-2021/027; date: 14 June 2021). As all larvae were euthanized by 5 days post-fertilization (dpf), approval from the Institutional Animal Care and Use Committee (QU-IACUC) was not required. Euthanasia was conducted in accordance with the policy set by the Ministry of Public Health and the American Veterinary Medical Association (AVMA) guidelines.

### 4.2. Experimental Design

To achieve the aims of this study, this project was divided into two major phases outlined by the following technical objectives: (1) to knock down the zebrafish orthologous gene, *gnas*, to mimic the loss of *GNAS* function in humans; and (2) to characterize the effects of *gnas* knockdown on various metabolic and developmental parameters in early larval zebrafish.

The first phase consisted of determining the optimal Morpholino (synthetic antisense oligonucleotide) dose that resulted in sufficient *gnas* knockdown with minimal toxic effects. To achieve this, one-cell-stage zebrafish embryos were divided into 5 groups of ~30 embryos as follows: (1) non-injected control group; (2) negative control group, injected with standard control Morpholino; (3–5) *gnas* knockdown groups injected with different doses (1, 3, and 5 ng, respectively) of the *gnas* Morpholinos. Western blotting was performed to quantify gene knockdown at the protein level, while Morpholino toxicity was assessed by measuring survival, hatching, and tail-flicking rates.

The second phase involved the functional evaluation of the dose-dependent effects of Morpholino-mediated *gnas* knockdown on various obesity and metabolism-related parameters in early larval zebrafish. The effective and minimally toxic Morpholino doses (3 and 5 ng) were selected for the functional verification of the knockdown, and the two control groups (non-injected and negative controls) were included for comparison. Here, the embryos or larvae were assessed at different timepoints for their survival and hatching rates, body length, yolk sac area, wet body mass, neutral lipid content (as determined by Oil Red O staining), metabolic rate (measured by the alamarBlue^TM^ assay), as well as levels of triglycerides, cyclic adenosine monophosphate (cAMP), and leptin using specialized enzyme-linked immunosorbent assay (ELISA) kits. All experiments were conducted with at least three replicates.

### 4.3. Morpholino Design and Preparation

Synthetic translation-blocking Morpholino antisense oligonucleotides (MOs) were designed by and purchased from Gene Tools, LLC (Philomath, OR, USA). The NCBI RefSeq accession numbers for the two target mRNA transcripts, encoded by the zebrafish orthologous gene, *gnas*, were submitted to Gene Tools via their online Morpholino design request form. The sequences of the obtained MO and the corresponding target mRNA accession numbers are listed in Table 1. Additionally, the Standard Control (Std Ctrl) MO was purchased as a negative control. This MO targets a specific β-thalassemia-causing intronic mutation in the human β-globin gene; thus, it has insignificant effects on phenotype in zebrafish and other model organisms. Prior to ordering, as per the recommendations of Gene Tools, the specificities of both experimental MOs were checked in silico using the NCBI Standard Nucleotide BLAST tool (BLASTN program), whereby the inverse complements of the MO sequences were entered as the nucleotide queries to identify any possible unintended targets in the zebrafish transcriptome.

The lyophilized MOs were each resuspended in autoclaved Milli-Q^®^ water to make stock solutions of 1 mM [80]. These stock solutions were stored at room temperature in their original vials, with caps tightly sealed using parafilm. For experimental use, working solutions were prepared by diluting the MO stock solutions as required, and phenol red was added as a tracer dye to a final concentration of 0.1% (*w*/*v*) in a final volume of 10 µL [80]. The two experimental MOs (*gnas*-MO1 and *gnas*-MO2) were combined in equal proportions to yield a total dose of 1, 3, and 5 ng of both MOs in an injection volume of 4.6 nL of each working solution (Table 2). Similarly, the Std Ctrl MO working solution was prepared to yield a final dose of 5 ng in each 4.6 nL injection volume. All working solutions were made in microcentrifuge tubes and stored at room temperature for subsequent use.

### 4.4. Microinjection Procedure

One-eight-cell-stage zebrafish embryos were microinjected into the yolk using the Drummond^TM^ Nanoject II Auto-Nanoliter Injector (Drummond Scientific Company, Broomall, PA, USA). Pulling of 3.5″ glass capillaries (Drummond Scientific Company, catalog # 3-000-203-G/X) was performed at 60 °C using a PC-100 Narishige puller (Narishige Group, Tokyo, Japan) to produce the micropipettes for injection. Under a stereomicroscope, the tips of the pulled micropipettes were broken to create narrow openings by lightly tapping them with the surface of a surgical blade [80]. The micropipettes were backfilled with mineral oil before drawing 1–2 µL of injection solution. Injection parameters were standardized across all experiments, with a volume set at 4.6 nL and an injection speed of 46 nL/s. The embryos were positioned in the grooves of a pre-prepared molded agarose block to stabilize them during injection [80]. The mold used for creating the grooves within the agarose substrate was purchased from World Precision Instruments (Z-MOLDS; WPI, Sarasota, FL, USA). Following injection, the embryos were transferred to a Petri dish with fresh egg water and incubated at 28.5 °C. Approximately two hours post-injection, the embryos were examined under the stereomicroscope to assess their viability. Any embryo found to be dead or exhibiting severe damage (e.g., dechorionated due to injection) was removed and excluded from the analysis.

### 4.5. Western Blotting

Zebrafish embryos or larvae were dechorionated (if unhatched) using pronase solution (2 mg/mL) and deyolked at the appropriate timepoint (48 h post-fertilization; hpf) for the verification of knockdown) as described in The Zebrafish Book [52]. The samples were then homogenized by sonication in Radioimmunoprecipitation Assay (RIPA) buffer (~3 µL/embryo) supplemented with protease and phosphatase inhibitors (Thermo Fisher Scientific, Waltham, MA, USA; catalog # 89900 and A32959). The homogenate was centrifuged at 15,000 RPM for 20 min at 4 °C, and the supernatant’s protein concentration was determined using the bicinchoninic acid (BCA) protein assay (Thermo Fisher Scientific, catalog # 23225). Equal amounts of protein (30–40 µg) were loaded onto 10% sodium dodecyl sulphate (SDS)-polyacrylamide gels and electrophoresis was performed. Subsequently, electrophoretic transfer of the proteins from the gels to polyvinylidene fluoride (PVDF) membranes (Thermo Fisher Scientific, catalog # LC2005) was conducted, after which the membranes were blocked for 1 h at room temperature in 5% non-fat dry milk in Tris-buffered saline (TBS) containing 0.05% Tween 20 (TBST; Glentham Life Sciences Ltd., Corsham, UK, catalog # GD9856). The blocked membranes were incubated overnight at 4 °C or for 2 h at room temperature with rabbit polyclonal anti-GNAS primary antibody (1:1000, Abcam, Cambridge, UK; catalog # ab97629). After washing thrice in TBST, the blots were incubated for 1 h at room temperature with horseradish peroxidase-conjugated anti-rabbit IgG secondary antibody (1:5000, Abcam, ab6721). The enhanced chemiluminescence (ECL) substrate (Thermo Fisher Scientific, catalog # 32106) and ChemiDoc MP Imaging System (Bio-Rad, Hercules, CA, USA; catalog # 12003154) were used to detect the protein bands. The blots were stripped with stripping buffer (Thermo Fisher Scientific, catalog # 21059), re-blocked, and re-incubated for 1 h at room temperature with anti-GAPDH antibody (1:5000, Abcam, ab210113) as the loading control, followed by secondary antibody incubation as described. SKB-F (human skeletal myoblasts, Zen-Bio, Durham, NC, USA) and A549 cell lysates were used as positive controls. Blot images were analyzed using ImageJ software (version 1.53t, Wayne Rasband, National Institute of Health, Bethesda, MD, USA) to quantify protein band densities.

### 4.6. Assessment of Survival, Hatching, and Tail-Flicking Rates

All experimental assessments were conducted using a ZEISS SterReo Disovery V12 stereomicroscope (ZEISS, Jena, Germany). The survival rates of zebrafish embryos or larvae were recorded daily from 24 hpf until the experimental endpoint. Survival was quantified as the percentage of embryos or larvae exhibiting a heartbeat, normalized to that of the non-injected control group. Hatching rates were recorded at 48 and 72 hpf, defined as the percentage of live embryos or larvae that had completely emerged from their chorion. The spontaneous tail-flicking rate was assessed at 24 hpf by recording 20 s videos (with a frame rate of 60 fps) of the embryos using the ORCA-Flash4.0 V3 camera (Hamamatsu Photonics K.K., Hamamatsu, Japan; catalog # C13440-20CU) mounted on a stereomicroscope along with the HCImage software (version 4.4.1.0, Hamamatsu Photonics K.K.), as described by Da’as et al. (2020) [39]. Subsequent analysis was performed using DanioScope software (version 1.1.110, Noldus Information Technology, Wageningen, The Netherlands), and the burst counts per minute (number of tail flicks per minute) were acquired.

### 4.7. Brightfield Imaging and Morphometric Measurements

Ten anesthetized larvae per group were assessed at 72 and 120 hpf for their body lengths and yolk sac areas. Brightfield images were captured using an ORCA-Flash4.0 V3 camera (Hamamatsu Photonics K.K., Hamamatsu, Japan) at 20X magnification under a ZEISS SteREO Discovery V12 stereomicroscope (ZEISS, Jena, Germany). Larvae were anesthetized by immersion in a solution of 2 µg/mL tricaine mesylate (MS-222). For imaging, a few drops of 3% (*w*/*v*) methylcellulose (Sigma-Aldrich, St. Louis, MO, USA; catalog # M0387) were placed in each cavity (~15 mm in diameter) of a triple cavity slide, and one or two larvae were laterally positioned with a probe in each cavity, straightened as much as possible, and the images were captured. After imaging, the larvae were transferred to fresh egg water and incubated at standard conditions to allow recovery.

ImageJ software (version 1.53t) was used to analyze the captured images. After setting the scale (pixels/mm), the body length was measured by drawing a straight line parallel to the larva’s full length (from the snout’s tip until the end of the caudal fin) using the ‘Straight Line’ selection tool. For yolk sac area measurement, the ‘Polygon’ area selection tool was used to trace the boundary of the entire yolk sac, including the yolk extension. The mean yolk sac area was normalized to that of the non-injected control group.

### 4.8. Larval Wet Body Mass Measurement

At 120 hpf, following imaging and prior to fixation, the wet masses of the larvae were measured using a precision scale with a resolution of 0.0001 g [70]. Larvae from each group were transferred to pre-weighed empty microcentrifuge tubes, and the egg water was removed as completely as possible. After weighing each tube with the larvae, the total mass of the larvae was calculated by subtracting the mass of the empty tube from each measurement. The average larval mass for each group was determined by dividing the total larval mass by the number of larvae in each tube.

### 4.9. Oil Red O Staining, Extraction, and Quantification in Zebrafish Larvae

Oil Red O (ORO) staining of zebrafish larvae was performed at 120 hpf to quantify the total amount of neutral lipids within the whole larval body, as described by Al-Jamal et al. (2020) and Yoganantharjah et al. (2017) [75,81]. Briefly, at 120 hpf, the larvae were fixed in 4% (*w*/*v*) paraformaldehyde in PBS (Thermo Fisher Scientific, Waltham, MA, USA; catalog # R37814) overnight at 4 °C. The fixed larvae were washed in 60% isopropanol before staining for 1.25 h in 0.25% (*w*/*v*) ORO (Abcam, Cambridge, UK; catalog # ab146295) solution. After washing the stained larvae in 60% isopropanol and 0.1% PBTw (PBS with 0.1% Tween 20), the larvae in each experimental or control group were divided into pools of 5–10 larvae per microcentrifuge tube, ensuring that the number was consistent across replicates and groups. Three to four replicates were analyzed per group. To extract the ORO stain from the zebrafish tissues, 250 µL of 4% (*v*/*v*) ethanol in 100% isopropanol was added to each microcentrifuge tube before incubation at room temperature for ~2 h or until the stain was completely extracted. To quantify the neutral lipid content, 200 µL of the solution in each tube was transferred to respective wells of a 96-well plate and the absorbance was measured at 495 nm using the Multiskan Sky Microplate Spectrophotometer (Thermo Fisher Scientific, catalog # 1530-800840). Fresh (unstained) 4% ethanol in isopropanol was used as a blank.

### 4.10. AlamarBlue^TM^ Zebrafish Metabolic Rate Assay

The alamarBlue^TM^ metabolic rate assay was conducted on zebrafish larvae as described in the literature [48,49]. Briefly, zebrafish larvae at 72 hpf from all experimental and control groups were rinsed once with sterile-filtered egg water before transferring to a 96-well plate. For each group, 3–5 wells, each containing 3 larvae, were included. The existing water in each occupied well was removed and replaced with 300 µL of assay buffer, composed of filtered egg water containing 1% alamarBlue™ HS Cell Viability Reagent (Thermo Fisher Scientific, Waltham, MA, USA; catalog # A50101), 0.1% dimethyl sulfoxide (DMSO), and 4 mM sodium bicarbonate. Two control wells containing only assay buffer were included as blank. The fluorescence of the plate was read promptly with excitation at 560 nm and emission at 590 nm, using the FLUOstar^®^ Omega microplate reader (BMG LABTECH, Ortenberg, Germany). The following endpoint settings were used: 10 flashes per scan point, 3 × 3 matrix scan, and 4 mm scan width. The plate was incubated in the dark at 28.5 °C for 24 h before reading the fluorescence again. The change in fluorescence was calculated after correcting for background fluorescence, and the data were normalized by setting the value of the non-injected control group to 1.

### 4.11. Measurement of Triglyceride, cAMP, and Leptin Levels in Larval Zebrafish Using ELISA

At 72 hpf, 30 to 40 whole zebrafish larvae from each group were pooled and homogenized as previously described. ELISA kits were purchased from Shanghai BlueGene Biotech (Shanghai, China) to measure triglyceride (catalog # E17T0018), cAMP (catalog # E17C0027), and leptin (catalog # E17L0038) levels in fish. A volume of homogenate containing 100 µg of total protein was made up in PBS to 100 µL and added to the appropriate coated well. The assay was performed as per the manufacturer’s instructions. Briefly, 100 µL of the standards and samples, assayed in duplicate, were incubated with 50 µL of conjugate for 1 h at 37 °C, followed by five manual washes. After washing, 50 µL of substrate A and 50 µL of substrate B were subsequently added to each well, and the plate was incubated for 20 min at 37 °C. Finally, 50 µL of stop solution was added to each well and the optical density was measured at 450 nm using the Multiskan Sky Microplate Spectrophotometer (Thermo Fisher Scientific, Waltham, MA, USA; catalog # 1530-800840). The assays were performed three times independently.

### 4.12. Statistical Analysis

Statistical analyses were performed using GraphPad Prism version 9.5.1 (GraphPad Software, San Diego, CA, USA). The results were analyzed using the one-way or two-way analysis of variance (ANOVA) tests with Tukey’s multiple comparisons test. All data were presented as mean ± standard error of the mean (SEM) of measurements from at least three independent experiments. A *p* value of <0.05 was considered statistically significant.

## 5. Conclusions

Inactivating genetic and epigenetic changes in *GNAS*, leading to G_s_α deficiency, are known to be associated with several disorders, including different variants of pseudohypoparathyroidism, which may feature severe, early-onset obesity as part of their clinical manifestations. Moreover, non-syndromic, monogenic obesity may result from such *GNAS* alterations. To expand our knowledge of the pathophysiology of this form of monogenic obesity, we developed the first zebrafish model of G_s_α deficiency. The zebrafish *gnas* morphants exhibited an obese phenotype characterized by significantly increased neutral lipid content, enlarged yolk sacs, and decreased metabolic rates, in addition to reduced body lengths and delayed hatching, mimicking the early-onset obesity, reduced energy expenditure, short stature, and developmental delay associated with PHP1A. This zebrafish model will facilitate future studies aiming to enhance our understanding of G_s_α-deficiency-associated early-onset obesity and its underlying mechanisms, potentially accelerating the discovery of novel targeted therapies for this form of obesity. In addition, it paves the way for efficient functional analysis of VUS and novel *GNAS* variants, improving diagnosis and patient care.

## Figures and Tables

**Table 1:** Morpholino oligomer (MO) sequences and their target mRNA transcripts in zebrafish.

MO Name	MO Sequence	Target mRNA Transcript ^1^
*gnas*-MO1	ACCAATGCTTGCTGTTTAACATCCG	XM_001335696.6
*gnas*-MO2	TCTTACTGTTGCCCAAACAACCCAT	XM_005172124.4
Standard Control	CCTCTTACCTCAGTTACAATTTATA	N/A

^1^ The NCBI RefSeq accession numbers for the mRNA transcripts are provided.

**Table 2:** Compositions of the MO working solutions.

Working Solution	Final MO Concentration in Injection Volume (mM) *	Volume of MO Stock Solution (µL)	Volume of Sterile MQ Water (µL)	Volume of 0.5% (*w*/*v*) Phenol Red (µL)
Expt MOs (1 ng)	0.026	0.13 (*gnas*-MO1)	7.74	2
		0.13 (*gnas*-MO2)		
Expt MOs (3 ng)	0.078	0.39 (*gnas*-MO1)	7.22	2
		0.39 (*gnas*-MO2)		
Expt MOs (5 ng)	0.13	0.65 (*gnas*-MO1)	6.70	2
		0.65 (*gnas*-MO2)		
Std Ctrl MO (5 ng)	0.13	1.31 (Std Ctrl)	6.69	2

* The molar masses of *gnas*-MO1, *gnas*-MO2, and Std Ctrl MOs (as indicated by Gene Tools) were 8394.04, 8323.01, and 8328 g/mol, respectively. The average molar masses of *gnas*-MO1 and *gnas*-MO2 were used for the calculations. Expt, experimental.

## Data Availability

The raw data supporting the conclusions of this article will be made available by the authors on request.
